# Posttranslational modification and mutation of histidine 50 trigger alpha synuclein aggregation and toxicity

**DOI:** 10.1186/s13024-015-0004-0

**Published:** 2015-03-11

**Authors:** Wei Xiang, Stefanie Menges, Johannes CM Schlachetzki, Holger Meixner, Anna-Carin Hoffmann, Ursula Schlötzer-Schrehardt, Cord-Michael Becker, Jürgen Winkler, Jochen Klucken

**Affiliations:** Institute of Biochemistry (Emil-Fischer-Center), Friedrich-Alexander-University of Erlangen-Nürnberg (FAU), Fahrstraße 17, 91054 Erlangen, Germany; Department of Molecular Neurology, University Hospital Erlangen, Friedrich-Alexander-Universität Erlangen-Nürnberg (FAU), 91054 Erlangen, Germany; Department of Ophthalmology, University Hospital Erlangen, Friedrich-Alexander-Universität Erlangen-Nürnberg (FAU), 91054 Erlangen, Germany

**Keywords:** Parkinson’s disease, Alpha synuclein, 4-hydroxy-2-nonenal, Posttranslational modification, Histidine 50, H50Q mutation

## Abstract

**Background:**

Aggregation and aggregation-mediated formation of toxic alpha synuclein (aSyn) species have been linked to the pathogenesis of sporadic and monogenic Parkinson’s disease (PD). A novel H50Q mutation of aSyn, resulting in the substitution of histidine by glutamine, has recently been identified in PD patients. We have previously shown that the lipid peroxidation product 4-hydroxy-2-nonenal (HNE) induces the formation of HNE-aSyn adducts, thereby promoting aSyn oligomerization and increasing its extracellular toxicity to human dopaminergic neurons. Intriguingly, we identified histidine 50 (H50) of aSyn as one of the HNE modification target residues. These converging lines of evidence support the hypothesis that changes in H50 via posttranslational modification (PTM) and mutation trigger the formation of aggregated, toxic aSyn species, which interfere with cellular homeostasis. In the present study, we aim to elucidate 1) the role of H50 in HNE-mediated aSyn aggregation and toxicity, and 2) the impact of H50 mutation on aSyn pathology. Besides the PD-related H50Q, we analyze a PD-unrelated control mutation, in which H50 is replaced by an arginine residue (H50R).

**Results:**

Analysis of HNE-treated aSyn revealed that H50 is the most susceptible residue of aSyn to HNE modification and is crucial for HNE-mediated aSyn oligomerization. Overexpression of aSyn with substituted H50 in H4 neuroglioma cells reduced HNE-induced cell damage, indicating a pivotal role of H50 in HNE modification-induced aSyn toxicity.

Furthermore, we showed *in vitro* that H50Q/R mutations substantially increase the formation of high density and fibrillar aSyn species, and potentiate the oligomerization propensity of aSyn in the presence of a nitrating agent. Cell-based experiments also revealed that overexpression of H50Q aSyn in H4 cells promotes aSyn oligomerization. Importantly, overexpression of both H50Q/R aSyn mutants in H4 cells significantly increased cell death when compared to wild type aSyn. This increase in cell death was further exacerbated by the application of H_2_O_2_.

**Conclusion:**

A dual approach addressing alterations of H50 showed that either H50 PTM or mutation trigger aSyn aggregation and toxicity, suggesting an important role of aSyn H50 in the pathogenesis of both sporadic and monogenic PD.

**Electronic supplementary material:**

The online version of this article (doi:10.1186/s13024-015-0004-0) contains supplementary material, which is available to authorized users.

## Background

Parkinson’s disease (PD) is the most prevalent neurodegenerative movement disorder worldwide. The disease is associated with a progressive loss of dopaminergic neurons in the *substantia nigra pars compacta*. An important neuropathological hallmark is the formation of eosinophilic inclusions called Lewy bodies (LB) and Lewy neurites in brains of PD patients, mainly consisting of aggregated alpha synuclein (aSyn), along with other proteins such as neurofilament subunits and ubiquitin [1]. The etiology of PD has not been fully understood. Environmental factors and genetic variants, which are proposed to affect neuronal function and eventually lead to degenerative processes, have been linked to an increased risk for PD. In particular, molecular and cellular disturbances including oxidative stress, mitochondrial impairment, and abnormal protein processing have been closely associated with PD [2].

Although the majority of PD cases are of unknown (sporadic) etiology, distinct genetic mutations of different genes account for approximately 10% of PD cases [3]. In particular, five point mutations in the coding sequence of the aSyn gene (SNCA) have been linked to autosomal dominant inherited PD, including the intensively studied A53T, A30P, and E46K mutations [4-6], and the very recently discovered H50Q and G51D mutations [7-9]. In addition, duplication and triplication of SNCA have been identified in familial forms of PD [10-12], suggesting that overexpression of the aSyn protein disturbs neuronal homeostasis. Furthermore, genome wide association studies revealed that genetic variants of SNCA are closely linked to sporadic PD [13,14]. The pathological relevance of aSyn in PD is further supported by the observation that aggregated and posttranslational modified aSyn is the major protein component in LB [15]. All these lines of evidence support a crucial role of aSyn, particularly of its aggregated forms, in the pathogenesis of PD.

Physiologically, aSyn is a soluble protein and is predominantly localized in neurons at presynaptic terminals in close proximity to synaptic vesicles [16]. aSyn aggregation is a process, in which the physiological soluble aSyn proteins change in conformation to produce pathological aggregated forms. Aggregated aSyn species include oligomers and fibrils, characterized by increased beta sheet content, insolubility, and detergent resistance [17]. Yet, it has not been clarified which factors trigger aggregation of aSyn and which aggregated aSyn species interfere with neuronal homeostasis in PD pathogenesis. Accumulating evidence supports that oxidative stress is an important modifier of aSyn aggregation. Oxidative stress is characterized by the accumulation of reactive oxygen/nitrogen species (ROS/RNS), which affect biomolecules, leading to posttranslational modifications (PTMs) of proteins and lipid peroxidation. Some of the generated aldehyde products during lipid peroxidation/degradation, such as 4-hydroxy-2-nonenal (HNE), are highly reactive to proteins and further trigger PTMs by forming aldehyde adducts on target proteins [18]. We have recently shown that ROS/RNS and HNE modify aSyn and promote the aggregation of aSyn [19]. Importantly, HNE modification of aSyn enhances the neurotoxicity of aSyn [20,21], in particular to human dopaminergic neurons [19]. Using mass spectrometry, we identified histidine 50 (H50) of aSyn as one of the target residues of HNE modification. Interestingly, a novel SNCA mutation, resulting in an amino acid exchange of H50 with a glutamine residue (H50Q), was recently reported in two PD patients [7,9]. These converging lines of evidence suggest that alterations of H50, via either PTM or genetic mutation, influence the aggregation propensity of aSyn and eventually induce cell damage.

To elucidate the relevance of H50 for aSyn pathology, in particular for aSyn aggregation and cytotoxicity, we investigated the role of H50 in 1) HNE-mediated, and 2) H50 mutation-induced aSyn pathology. In addition to H50Q, we analyzed a PD-unrelated mutation in order to answer the question of whether the potential changes induced by the PD-related H50Q mutation are due to the loss of H50, or are attributed to the specific effect of the glutamine residue. To this end, we replaced H50 by an arginine residue (H50R), which belongs to the group of polar and basic amino acids like histidine. Our results show that both posttranslational and genetic modifications of aSyn at the specific H50 residue increase the aggregation propensity and toxicity of aSyn.

## Results

### aSyn H50 is the most reactive residue for HNE modification

HNE, a lipid peroxidation product, reacts with histidine, cysteine, and lysine residues of proteins, leading to the formation of HNE-protein adducts [18]. Since aSyn does not have cysteine residues, the only histidine residue (H50) and 15 lysine residues are potential targets for HNE modification. Recently, we showed that H50 of aSyn is a target residue for HNE modification, although it is not the only possible modification site in aSyn [19]. Indeed, HNE modifications of Lys 60 and Lys 96 have also been described [22]. Thus, our first aim was to elucidate the role of H50 for HNE modification in aSyn.

**Figure 1:**
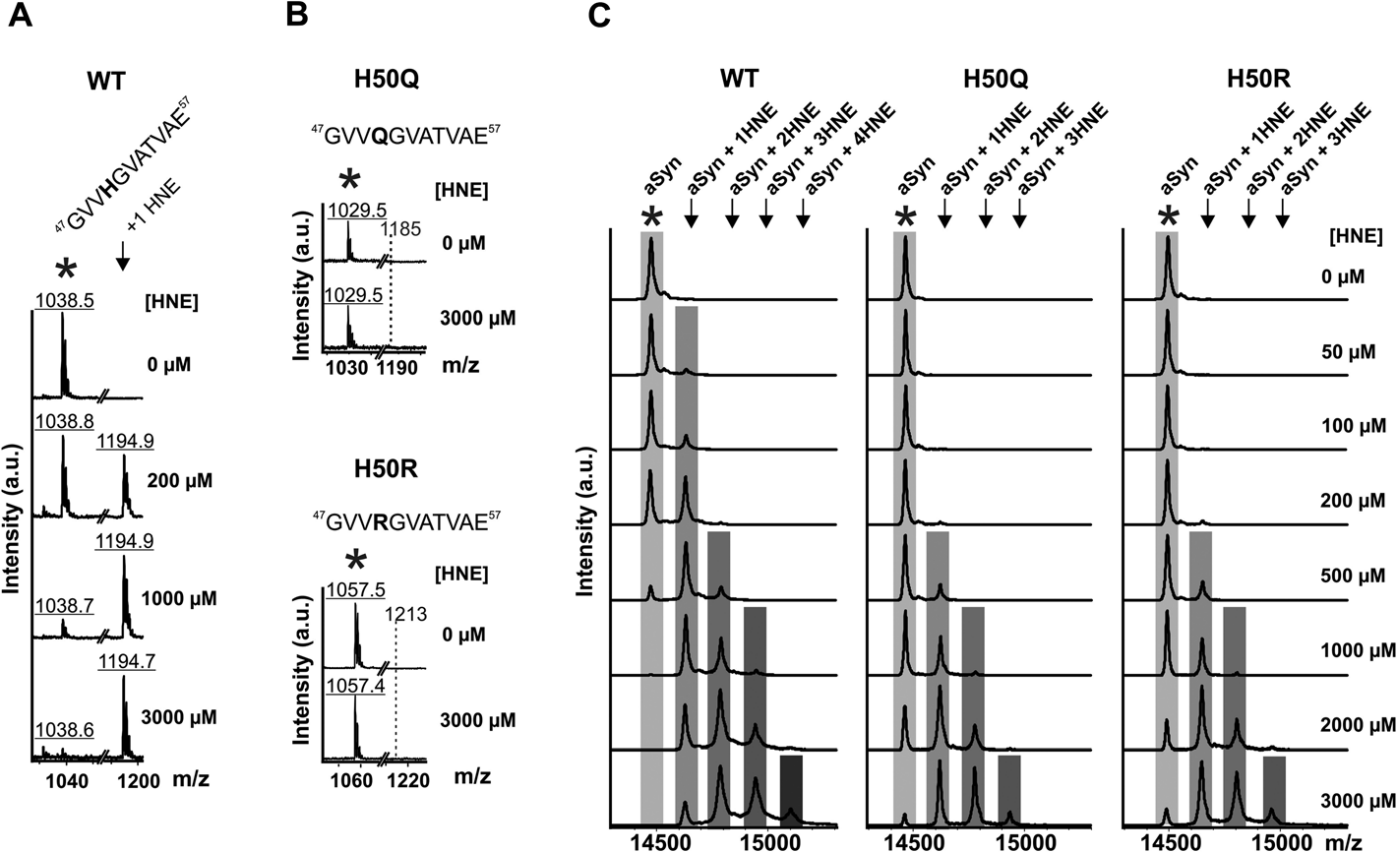
**HNE modification of WT and H50 mutant aSyn. A)** Recombinant WT aSyn treated with different concentrations of HNE (0 - 3000 μM) for 24 h was digested by GluC in order to measure HNE modification of the H50 containing peptide ^47^GVV**H**GVATVAE^57^ by MALDI-TOF analysis. The modification of this peptide (*observed monoisotopic mass to charge ratio m/z: 1038) by HNE leads to a mass increase of 156 Da (↓ observed monoisotopic m/z: 1194). **B)** aSyn H50Q and H50R mutations completely abolish HNE modification of the corresponding peptides containing the residue 50. Only unmodified peptides, i.e. ^47^GVV**Q**GVATVAE^57^ (*observed monoisotopic m/z: 1029) of the H50Q aSyn and ^47^GVV**R**GVATVAE^57^ (*observed monoisotopic m/z: 1057) of the H50R aSyn, are observed. **C)** Recombinant full-length WT and H50Q/R aSyn were treated with different HNE concentrations for 24 h and analyzed by MALDI-TOF MS. Unmodified (*) and HNE-modified (↓) aSyn (WT, H50Q, and H50R) with a characteristic mass difference of 156 Da are indicated. H50Q/R mutations reduce the formation of aSyn-HNE-adducts, particularly they prevent the modification by HNE at low concentrations (50 - 200 μM).

We applied site-directed mutagenesis to exchange the H50 residue and to produce two recombinant aSyn H50 mutants, i.e. H50Q and H50R. HNE concentration ranges *in vivo* from 0.1 - 3 μM under physiological conditions and may increase up to 10 - 5000 μM under pathological conditions of increased oxidative stress [18,23]. In order to analyze the reactivity of H50 to HNE, we incubated recombinant wild type (WT) and H50Q/R mutant aSyn with pathologically relevant HNE concentrations (50 – 3000 μM). The addition of one HNE molecule to a target amino acid residue is characterized by a mass increase of 156 Da. Matrix-assisted laser-desorption-ionization time-of-flight mass spectrometry (MALDI-TOF MS) analysis of GluC-digested WT aSyn exposed to HNE revealed HNE modification of the H50 containing peptide ^47^GVV**H**GVATVAE^57^ (Figure 1A). The shift from unmodified (*) to modified peptide (↓) increased in a HNE concentration-dependent manner. Both H50 mutants (H50Q/R) completely abolished HNE modification of the corresponding residue 50 containing peptides (Figure 1B).

MALDI-TOF MS analysis of full-length aSyn revealed that HNE addition to WT aSyn is already detectable at a HNE concentration of 50 μM (Figure 1C). Incubation of WT aSyn with HNE at low concentrations (50 - 200 μM) resulted in the addition of a single HNE molecule. HNE concentrations from 500 to 3000 μM induced the formation of additional HNE adducts in WT aSyn, indicating the existence of more than one modifiable amino acid residue at high HNE concentrations. In contrast to WT aSyn, HNE adducts were barely detectable in aSyn H50 mutants (H50Q/R) exposed to low HNE concentrations (50 - 200 μM). Only high concentrations of HNE applied to H50 mutant aSyn led to the formation of HNE adducts. This result revealed that other modifiable residues of aSyn (e.g. lysine residues) exhibit a lower reactivity to HNE and thus indicate that H50 is the initial target residue of HNE modification.

### aSyn H50 is the crucial residue for HNE-mediated oligomerization

**Figure 2:**
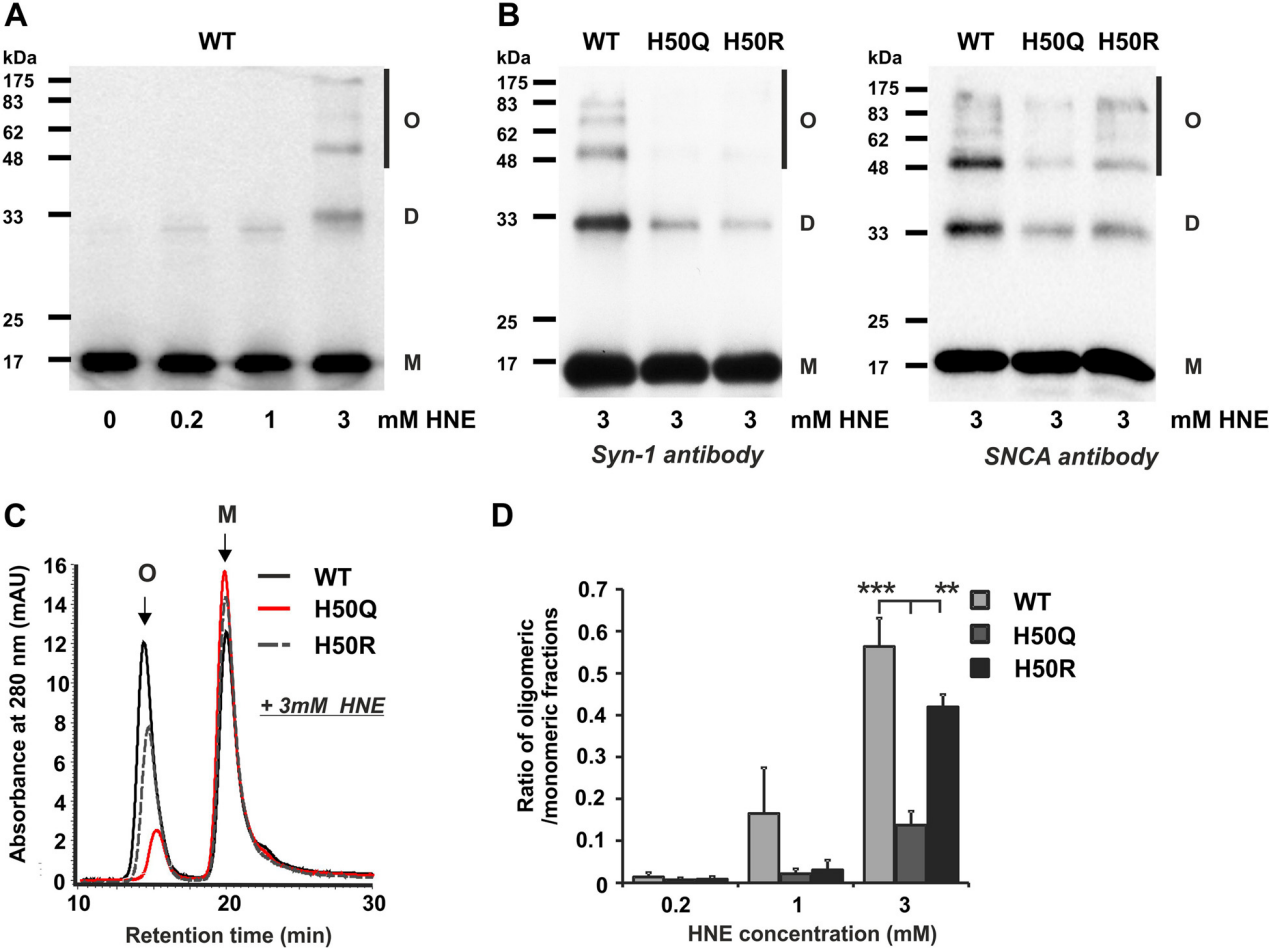
**HNE-induced aggregation of WT and H50 mutant aSyn. A)** Recombinant human WT aSyn was treated with 0, 0.2, 1, and 3 mM HNE for 24 h and analyzed by SDS-PAGE followed by WB using an antibody against aSyn (Syn-1). The formation of SDS-resistant aSyn oligomers as indicated shows an increase in a HNE-concentration dependent manner. M: aSyn monomers; D: aSyn dimers; O: aSyn oligomers. **B)** Recombinant human WT and H50Q/R aSyn were treated with 3 mM HNE for 24 h and analyzed by SDS-PAGE and WB. H50Q/R mutations decrease the formation of HNE-induced SDS-resistant aSyn oligomers as probed by two different antibodies raised against distinct aSyn antigenes (Syn-1 antibody, left, and SNCA antibody, right). **C)** WT, H50Q, and H50R aSyn were treated with 3 mM HNE for 24 h and subsequently analyzed by SEC in order to assess the formation of soluble oligomers. aSyn monomers (M) and oligomers (O) are distinguished by different retention times. **D)** Area ratio of oligomeric to monomeric fraction in HPLC chromatograms of SEC was calculated for aSyn incubated with 0.2, 1, or 3 mM HNE. H50 mutations reduce the formation of HNE-induced soluble oligomers. Significance was determined by a one way ANOVA Tukey’s Multiple Comparison Test. n = 3. Error bars = SEM.

HNE triggers the oligomerization of aSyn [19]. As lysine residues may also be involved in HNE modification, we asked whether HNE modification of H50 is the major factor for HNE-mediated oligomerization. We exposed recombinant human WT and H50Q/R aSyn to different HNE concentrations and investigated the HNE-mediated oligomerization by SDS-PAGE followed by Western blot (WB) analysis (Figure 2A and B) and size exclusion chromatography (SEC) (Figure 2C and D). We observed that SDS-resistance of aSyn oligomers is dependent on the applied HNE concentration. SDS-resistant oligomers of WT aSyn were detectable after exposure to 3 mM HNE (Figure 2A). Importantly, at the same HNE concentration, i.e. 3 mM, H50 mutations reduced the formation of HNE-induced, SDS-resistant aSyn oligomers (Figure 2B). This effect was reproducibly detected using different antibodies raised against distinct aSyn antigens. Using SEC analysis, we observed an increase in the formation of soluble WT aSyn oligomers after HNE exposure (Figure 2C). In accordance to the results of SDS-PAGE, H50 mutations, in particular H50Q, also decreased the formation of HNE-mediated soluble oligomers (Figure 2D). While SDS-PAGE detects SDS-resistant oligomers (both soluble and insoluble), SEC examines soluble oligomers (both SDS-resistant and non-resistant). Both approaches provided consistent results, indicating that H50 is the initial factor contributing to HNE-induced oligomerization of aSyn.

### The substitution of aSyn H50 reduces HNE-induced cytotoxicity

**Figure 3:**
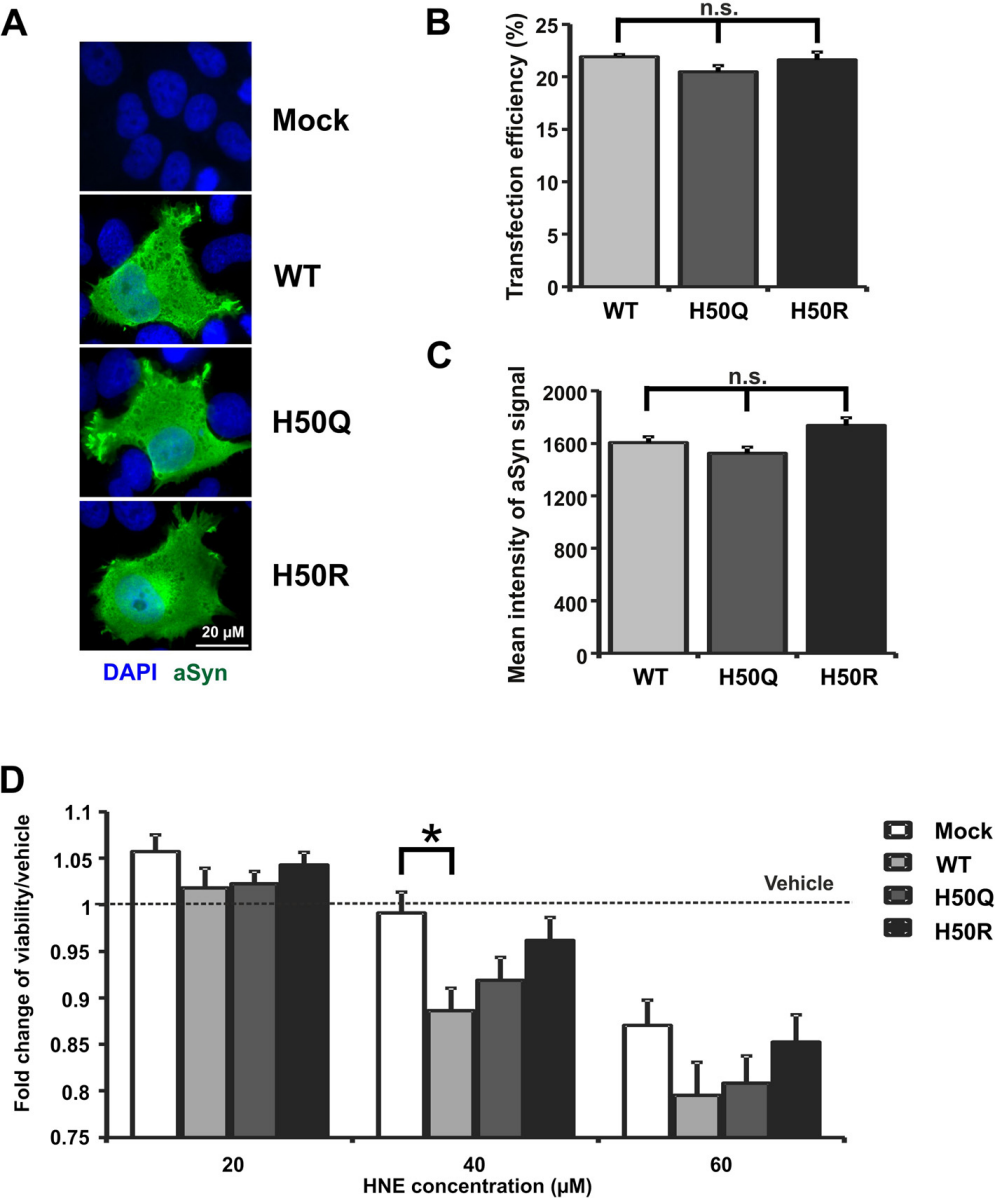
**HNE-induced cytotoxicity in WT and H50 mutant aSyn overexpressing cells. A)** Expression of aSyn in H4 cells transiently transfected with WT aSyn and H50 mutants (H50Q/R) was confirmed by ICC using an antibody against human aSyn (green) and counterstaining by DAPI (blue). **B)** Transfection efficiency and **C)** aSyn expression levels in H4 cells transfected with WT aSyn and H50 mutants were assessed via flow cytometry at the time point of HNE treatment (24 h posttransfection). WT and H50Q/R aSyn transfected cells show an equal proportion of aSyn overexpressing cells **(B)** as well as comparable aSyn expression levels **(C)**. Significance was determined by one way ANOVA Tukey’s Multiple Comparison Tests. n = 3. Error bars = SEM. **D)** H4 cells transiently transfected with mock, WT, and H50Q/R aSyn were treated with different HNE concentrations (20 – 60 μM) for 12 h and cellular viability was measured by MTS assay. HNE induces a dose-dependent reduction of cell viability. H50Q/R mutant aSyn transfected cells appear to be more resistant to HNE than WT aSyn transfected cells. Significance was determined by two way ANOVA Bonferroni Multiple Comparisons. n = 8. Error bars = SEM.

HNE modification increases the cytotoxicity of aSyn when applied to cells extracellularly [19]. Furthermore, HNE modification of intracellularly overexpressed aSyn was shown in cells exposed to extracellular HNE [22]. Thus, we asked whether H50, the most reactive target residue of aSyn for HNE modification (as described above, Figure 1), influences the susceptibility of aSyn overexpressing cells to HNE exposure. To address this question, we generated pcDNA3.1 plasmids encoding human WT or H50Q/R mutant aSyn for transient transfection in mammalian cells. To reduce the interference between endogenous and overexpressed aSyn variants, we chose human H4 neuroglioma cells, which only express very low levels of endogenous aSyn. aSyn overexpression was confirmed via immunocytochemistry (ICC) and flow cytometric analysis (Figure 3A, B and C). The proportion of aSyn transfected cells showed a time dependent increase, reaching 39 - 48% 36 h after transfection (Additional file 1: Figure S1). H4 cells transfected with WT, H50Q, and H50R aSyn vectors showed a comparable transfection rate for all measured time points (17 – 36 h posttransfection, Additional file 1: Figure S1C). No significant differences in both transfection efficiency and aSyn expression levels were detected 24 h after transfection, when we started the treatment experiments (Figure 3B and C). We incubated WT and H50Q/R mutant aSyn, as well as mock transfected cells, with different concentrations of HNE and measured cell damage by using MTS viability assay. While HNE exposed WT aSyn overexpressing cells showed a reduced viability compared to mock-transfected cells for the HNE concentrations used (20 – 60 μM), in particular for 40 μM HNE, cell damage of H50Q and H50R overexpressing H4 cells exposed to the same HNE concentrations was less pronounced (Figure 3D). The complementary flow cytometric analysis of transfected H4 cells treated with HNE supported that H50 mutant aSyn overexpressing cells, in particular the aSyn H50R overexpressing cells, are more resistant to HNE exposure than WT aSyn transfected cells (Additional file 1: Figure S2). These results suggest that the exchange of the H50 residue attenuates the toxic effect of HNE, supporting the essential role of H50 HNE modification for HNE-induced cytotoxicity.

### H50 mutations increase the aggregation propensity of aSyn both *in vitro* and in cells

Modification of H50 is crucial for HNE-induced aSyn oligomerization. Thus, we asked whether the alteration of H50 through genetic point mutation, particularly the novel reported H50Q mutation, also influences the aggregation propensity of aSyn. In addition to H50Q, we analyzed a non PD-related control mutation (H50R) to specify whether changes caused by H50Q mutation are due to the loss of H50 or attributed to the specific effect of the glutamine residue.

**Figure 4:**
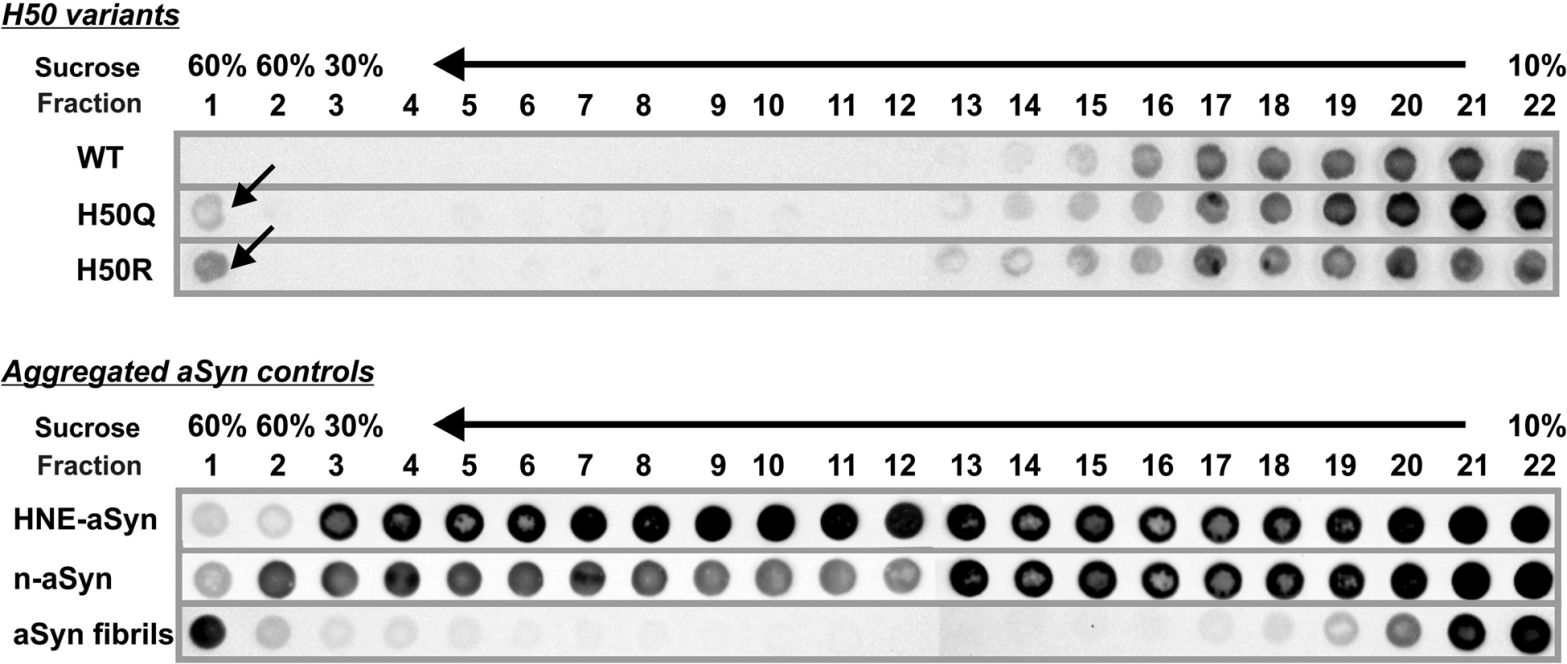
**SDGC analysis of recombinant WT, H50Q, and H50R aSyn.** Recombinant human WT aSyn is clearly detected in the low density factions from 16 to 22 (row 1 of the upper panel). Oligomerized aSyn is characterized by a signal shift towards higher density fractions, which is exemplarily displayed by HNE-aSyn and n-aSyn consisting of oligomeric aSyn (rows 1 and 2 of the lower panel). Large fibrillar aggregates appear in the first exclusion fractions (row 3 of the lower panel). H50 mutations (H50Q and H50R) increase aSyn immunosignal in the 60% high density exclusion fraction (arrows, rows 2 and 3 of the upper panel).

To evaluate the intrinsic aggregation propensity of mutant H50 variants, we applied sucrose density gradient centrifugation (SDGC), and used the collected fractions for dot blot analysis in order to detect aggregation of aSyn. To optimize the separation range of the density gradient, we first analyzed control samples including WT monomeric aSyn (WT, Figure 4, upper panel), highly oligomerized HNE-modified WT aSyn (HNE-aSyn, Figure 4 lower panel), nitrated WT aSyn (n-aSyn, Figure 4 lower panel) as characterized previously [19], and WT aSyn amyloid-like fibrils prepared after agitating aSyn (Figure 4, lower panel). By using a sucrose gradient of 10 - 30% and an exclusion layer (60% sucrose) under the gradient, WT aSyn monomers were clearly detected in the low density factions 16 - 22. HNE-aSyn and n-aSyn containing oligomers showed a shift of aSyn positive fractions to higher density fractions. Large aSyn aggregates like aSyn fibrils were observed in the first exclusion fractions. We next compared the aSyn positive fractions after SDGC analysis of recombinant WT and aSyn H50 mutants prepared from *E. coli*. In contrast to WT aSyn, a proportion of aSyn H50 mutants was detected in the first exclusion fraction with a high sucrose density (60%), indicating an increased intrinsic aggregation propensity of H50Q/R aSyn (Figure 4, upper panel).

**Figure 5:**
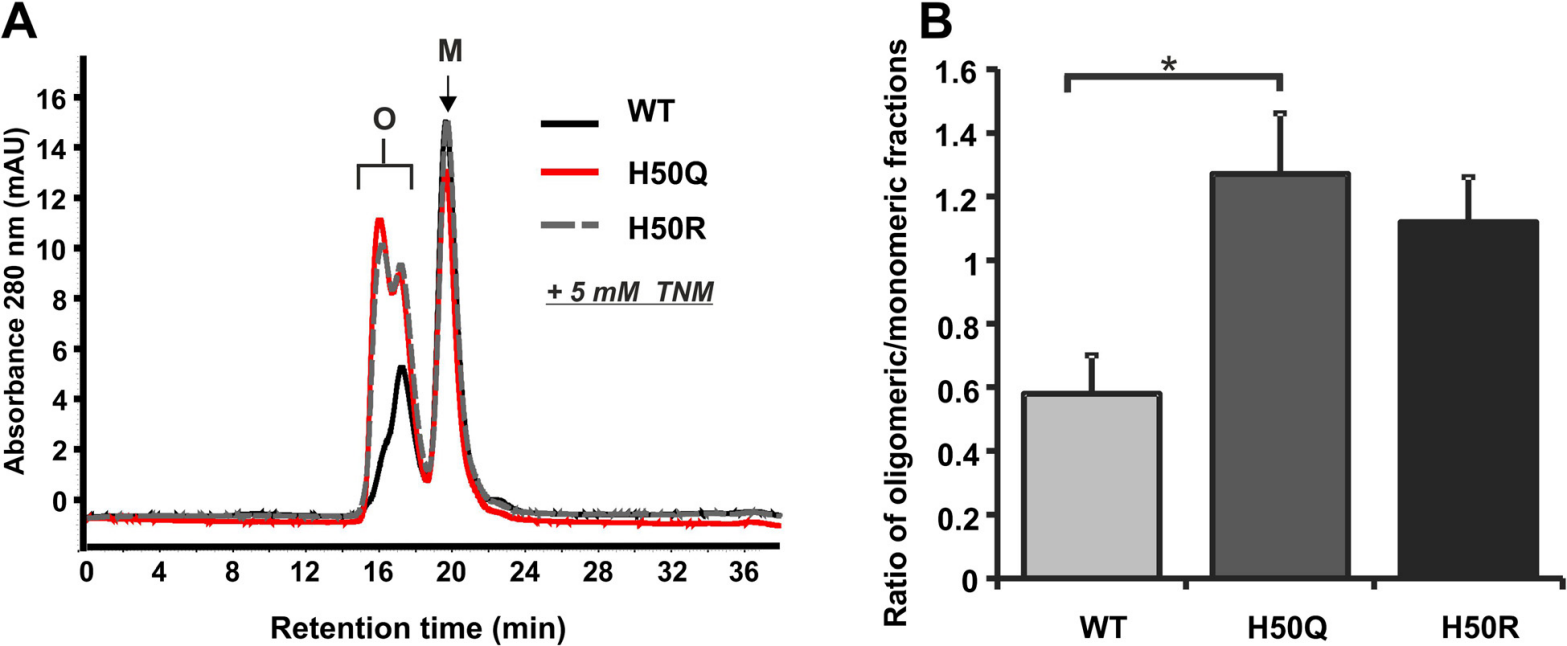
**Nitration-induced oligomerization of WT aSyn and aSyn H50 mutants. A)** Recombinant WT aSyn and aSyn H50 mutants were treated with 5 mM TNM for 7 h to induce nitration-mediated oligomerization of aSyn. Formation of soluble aSyn oligomers after TNM treatment was assessed by SEC. aSyn monomers (M) and oligomers (O) are distinguished by different retention times. **B)** Quantification of soluble oligomers was performed by calculating the area ratio of oligomeric fraction to monomeric fraction. H50 mutations increase the formation of soluble oligomers after TNM treatment. Particularly, the H50Q mutation significantly enhances the propensity of nitration-mediated aSyn oligomerization. Significance was determined by a one way ANOVA Tukey’s Multiple Comparison Test. n = 3. Error bars = SEM.

To assess the potential existence of soluble, oligomeric species in recombinant aSyn samples, we further analyzed WT and H50 mutant aSyn by using SEC. We did not detect an intrinsic formation of soluble oligomers in untreated WT aSyn and in both H50 aSyn mutants (data not shown). Our previous studies have shown that increased ROS/RNS induce the oxidation/nitration of aSyn. In particular, nitration of aSyn significantly promotes the formation of soluble aSyn oligomers [19]. Thus, we asked whether nitration-mediated aSyn oligomerization is influenced by H50 mutations. Nitrated aSyn was generated by incubating WT and H50Q/R aSyn with tetranitromethane (TNM), an efficient nitrating agent [19]. Nitration triggered the formation of soluble oligomers, which were readily detected in WT aSyn as well as in H50Q/R mutants (Figure 5A). Notably, the H50Q mutation significantly increased the level of TNM-induced oligomeric aSyn compared to WT aSyn (Figure 5B). The H50R mutation also increased nitration-induced oligomerization of aSyn, albeit without reaching statistical significance. This finding demonstrates that H50 mutations exacerbate nitration-mediated oligomerization, suggesting an increased oligomerization of aSyn H50 mutants under oxidative stress conditions.

**Figure 6:**
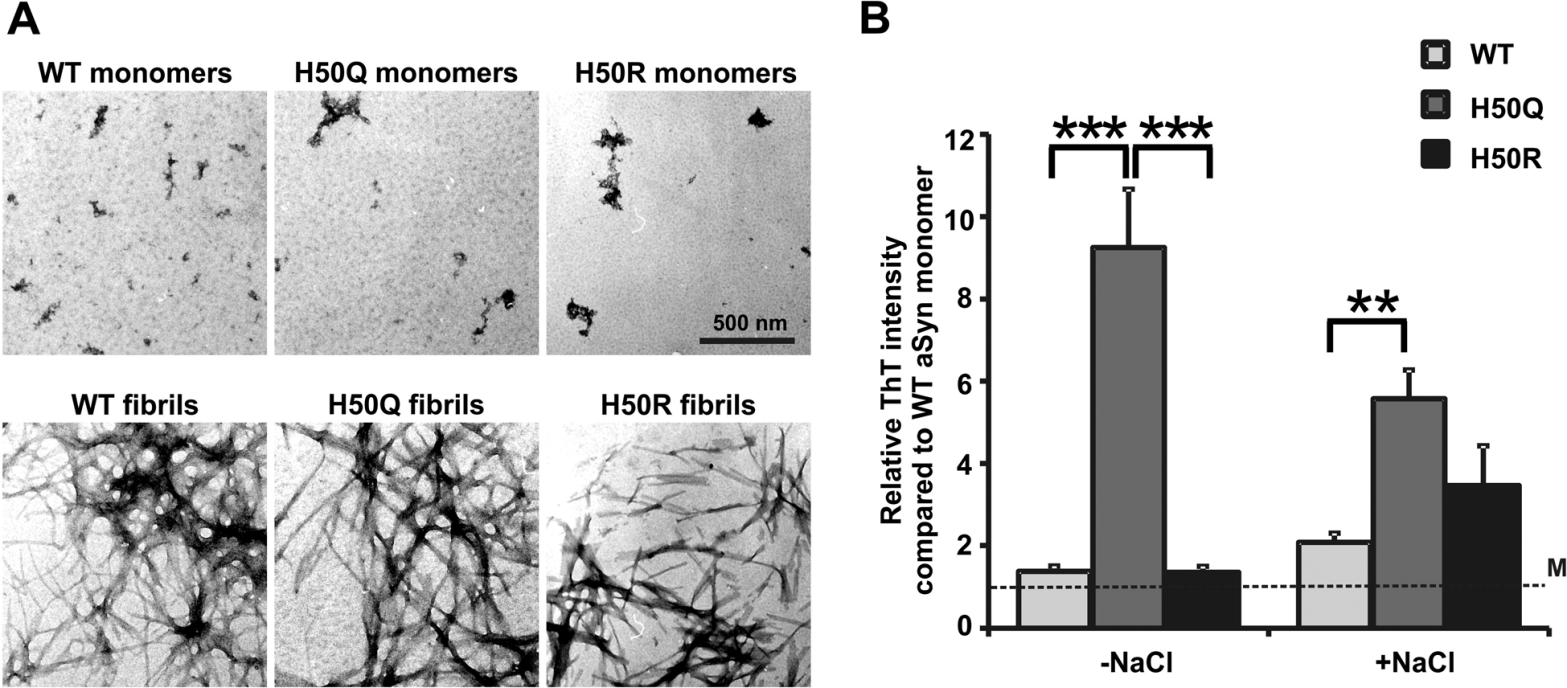
**Fibrillization of WT and H50 mutant aSyn. A)** EM analysis of fibrils derived from recombinant WT and H50Q/R aSyn agitated for 7 days. **B)** Recombinant WT and H50Q/R aSyn were agitated for 7 days with (+NaCl) and without (-NaCl) 160 mM NaCl. The formation of aSyn fibrils was assessed by ThT assay. ThT intensity was normalized to non-agitated, monomeric WT aSyn (M, dashed line). H50Q aSyn mutation significantly increases the formation of aSyn fibrils with and without NaCl. Significance was determined by one way ANOVA Tukey’s Multiple Comparison Tests. n = 6 (-NaCl), n = 8 (+NaCl). Error bars = SEM.

In order to study the propensity of H50 mutants to form amyloid fibrils, we generated aSyn fibrils by agitating WT and H50Q/R mutant aSyn at 37°C for 7 days. Electron microscopy (EM) analysis revealed that WT and H50Q/R aSyn form fibrillar structures after agitation (Figure 6A). Assessment of amyloid fibrils using Thioflavin T (ThT) assay demonstrated that H50Q aSyn significantly increases fibrillization compared to WT aSyn both in the absence or presence of NaCl (Figure 6B). The influence of the H50R mutation on aSyn fibrillization was dependent on the salt concentration in the agitation buffer. In the presence of 160 mM NaCl at a physiological ionic strength the H50R mutant also showed an increase in fibrillization of aSyn, although not reaching statistical significance.

**Figure 7:**
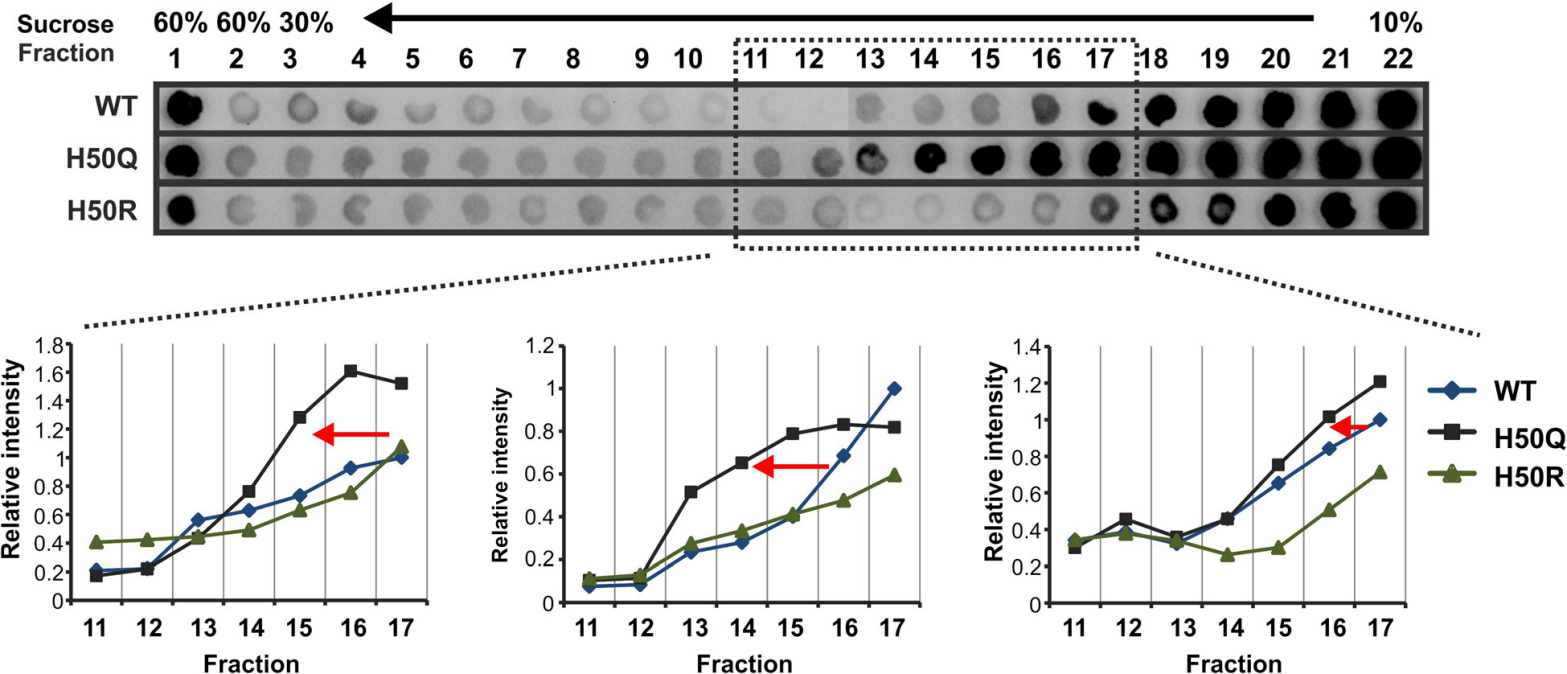
**Intracellular aggregation of WT and H50 mutant aSyn.** H4 neuroglioma cells were transfected with WT and H50Q/R mutant aSyn. SDGC analysis of lysates from H4 cells transfected with WT and H50Q/R mutant aSyn was performed. A shift of aSyn positive fractions from lower density fractions to higher density fractions was observed for the aSyn H50Q mutant. The quantification of aSyn immunointensity in fractions 11 - 17 in 3 independent experiments is shown in the lower panel. To allow comparison, all fractions collected from WT and H50Q/R mutants in each experiment were loaded on the same dot blot membrane. The relative aSyn intensity was generated by normalizing the intensity of each fraction to the intensity of the fraction 17 from WT aSyn in each experiment.

Complementary assessments of aSyn aggregation *in vitro* revealed that compared to WT aSyn, H50 mutant aSyn is more prone to form 1) high density aggregates (SDGC), 2) soluble oligomers after exposure to a nitrating agent (SEC), and 3) amyloid fibrils after agitation (ThT assay), suggesting a general enhanced aggregation propensity due to H50 mutations. Thus, we asked if H50 mutations also increase the aggregation propensity of intracellularly expressed aSyn. To this end, we transiently transfected H4 cells with WT and H50Q/R aSyn constructs. The lysates of transfected cells were analyzed by SDGC followed by dot blot analysis. In the lysates of H50Q aSyn transfected cells, we observed a shift of aSyn immunoreactivity from lower to higher density fractions compared to WT aSyn, particularly in fractions 11 - 17 (Figure 7). Quantification of aSyn immunointensity from independent analyses revealed a reproducible increase in the aSyn signal in these fractions of higher density for H50Q aSyn samples, confirming an increased oligomerization propensity of cellularly expressed H50Q mutant aSyn (Figure 7 lower panel). In contrast to the H50Q mutant, the H50R mutant did not show an obvious increase in the aSyn signal in the higher density fractions 11 - 17.

### Overexpression of H50 mutant aSyn increases cell damage

**Figure 8:**
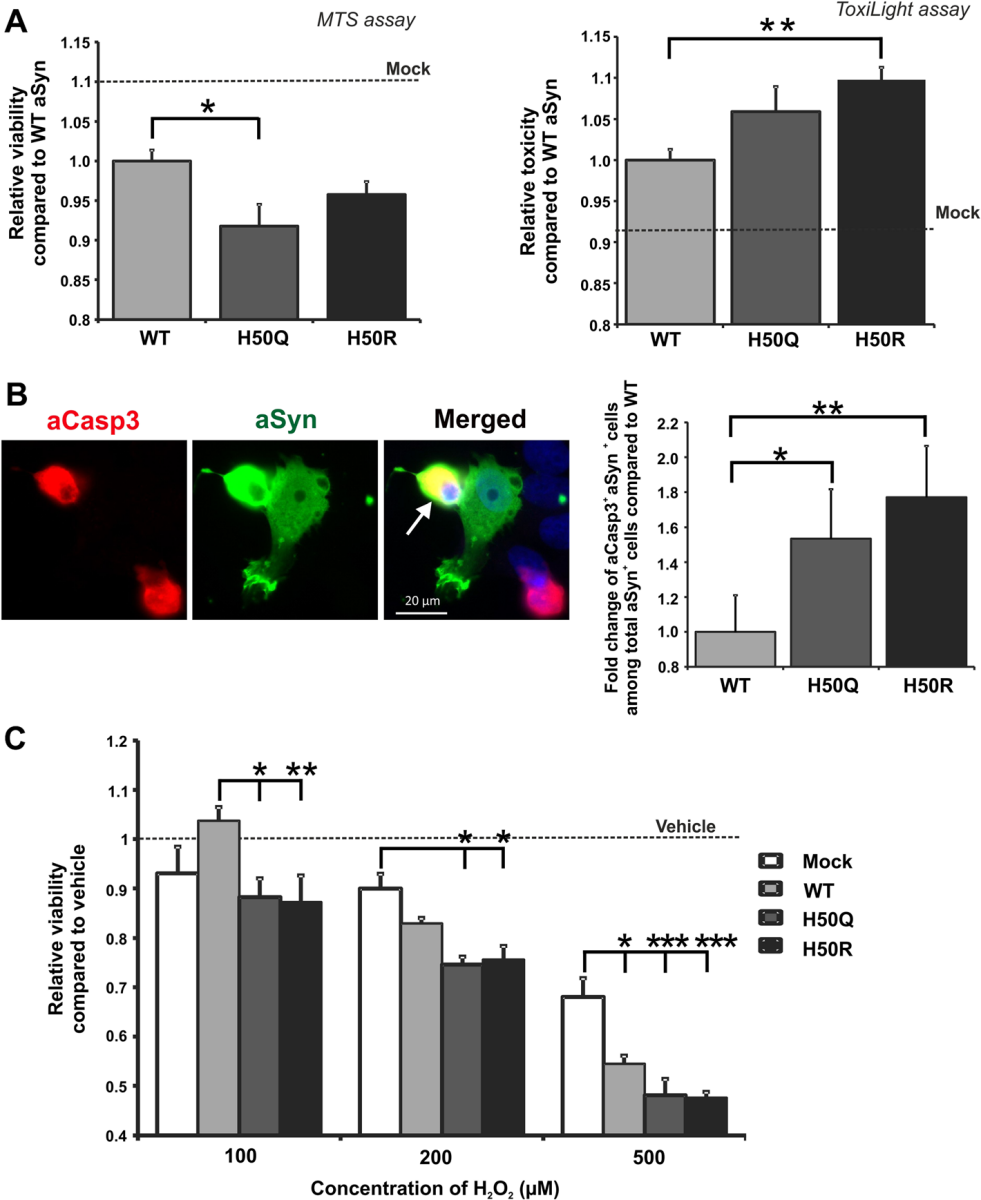
**Toxicity of intracellularly overexpressed WT and H50 mutant aSyn. A)** H4 neuroglioma cells were transiently transfected with mock, WT, and H50 mutant aSyn, and cell damage was assessed by using MTS cell viability (left) and ToxiLight assays (right). For quantification, measured values were normalized to the mean of WT samples. The background levels of viability and toxicity of mock transfected cells are indicated as dashed lines. Both assays show a slight increase in general cell damage in cells transfected with either H50Q or H50R aSyn. Significance was determined by a one way ANOVA Tukey’s Multiple Comparison Test for randomized block experiments. n = 8 (ToxiLight) and n = 12 (MTS assay). Error bars = SEM. **B)** The induction of apoptosis by overexpressed H50 mutant aSyn was assessed by ICC using antibodies against aSyn (green) and activated Caspase 3 (aCasp3, red). The number of apoptotic, (aCasp3+ aSyn+, arrow) out of total aSyn overexpressing cells (aSyn+) was counted. The proportion of apoptotic cells in H50Q/R aSyn overexpressing cells was significantly higher than in WT aSyn overexpressing cells. For quantification, measured values were normalized to the mean of WT samples. Significance was determined by a one way ANOVA Tukey’s Multiple Comparison Test for randomized block experiments. n = 6. Error bars = SEM. **C)** H4 cells transfected with mock, WT, and H50 mutant aSyn were treated with H_2_O_2_ 24 h after transfection for another 24 h followed by MTS viability assay. The viability of treated cells was compared to the viability of the corresponding untreated cells (vehicle, dashed line). H_2_O_2_ treatment induces a more pronounced reduction of the viability of H50Q/R overexpressing cells than of WT overexpressing cells. Significance was determined by two way ANOVA Bonferroni Multiple Comparisons. n ≥ 3. Error bars = SEM.

To elucidate whether mutation of H50 increases cytotoxicity of aSyn, we transiently transfected H4 cells with WT and H50Q/R aSyn constructs. Cell damage was assessed by MTS viability assay and ToxiLight toxicity assay. H4 cells transiently transfected with H50 aSyn mutants showed a slight reduction in cell viability and an increase in cell death compared to H4 cells transfected with WT aSyn (Figure 8A). As viability and toxicity assays such as MTS and ToxiLight only determine general damage of transiently transfected cells including overexpressing and non-overexpressing cells, we also used ICC to detect apoptosis specifically in aSyn overexpressing cells (Figure 8B). The number of cells double positive for aSyn and activated (cleaved) Caspase 3 (aCasp3) (aSyn+/aCasp3+) among all aSyn+ cells was determined. Overexpression of H50Q/R mutants led to a significant increase in the ratio of cells double positive for aCasp3 and aSyn as compared to the overexpression of WT aSyn, indicating that intracellular overexpression of H50 mutants promotes apoptosis (Figure 8B).

Since our results showed that overexpression of H50Q/R mutant aSyn attenuates cell susceptibility to HNE as compared to WT aSyn (Figure 3, Additional file 1: Figure S2), we asked whether overexpression of H50 mutant aSyn would differently impact the response of cells to another oxidative stressor, e.g. H_2_O_2_. Therefore, we analyzed the influence of overexpression of WT and H50Q/R mutant aSyn on cell viability in the presence of H_2_O_2_, a global inducer of oxidative stress. Application of H_2_O_2_ induced a concentration-dependent decrease in the viability of H4 cells transfected with WT and H50Q/R mutant aSyn (Figure 8C). In order to focus on the potential differences in the susceptibility to H_2_O_2_ and thus, to eliminate baseline toxic effects induced by overexpression of H50 mutant aSyn, the fold reduction in the viability of transfected cells was calculated compared to the corresponding vehicle-treated cells. We observed that the H_2_O_2_ induced decline in cell viability was generally more pronounced in H50Q/R aSyn than in WT aSyn transfected cells at a H_2_O_2_ concentration range of 100 – 500 μM. This effect was statistically significant when using a lower H_2_O_2_ concentration of 100 μM. This finding suggests that H50 mutations increase the cellular susceptibility to oxidative stress.

## Discussion

In the present study, we used a dual approach to alter the H50 residue of aSyn, i.e. HNE-PTM and DNA mutation-induced H50 substitution. We provide an *in vitro* and cell-based characterization of the impact of these alterations on aSyn aggregation and toxicity, two important features of aSyn pathology, which are crucial for the pathogenesis of PD.

The relevance of HNE modification of aSyn in the pathogenesis of PD is strengthened by several lines of evidence: 1) Oxidative stress and lipid peroxidation are strongly associated with neurodegeneration in PD. HNE along with other reactive aldehyde products, such as glyoxal, malondialdehyde, and 4-oxo-2-nonenal (ONE), are important products of lipid peroxidation [18]; 2) The level of HNE-modified proteins is enhanced in affected brain areas, particularly in aSyn containing LBs in PD and other neurodegenerative disorders with LB pathology [24-26]; 3) Several studies have shown that HNE readily modifies aSyn *in vitro* and in cells exposed to HNE [19,20,22]; and 4) HNE modification not only increases aggregation propensity of aSyn *in vitro*, but HNE-modified aSyn also provokes neuronal damage when applied extracellularly to neurons [19-21]. However, the role of the different HNE target sites of aSyn in these HNE-mediated effects (e.g. aSyn modification, aggregation, and toxicity) has not yet been clarified. Among the possible target sites of HNE, H50 is of particular interest, because a novel aSyn point mutation affecting this residue (H50Q) has recently been reported in monogenic PD patients.

To elucidate the role of H50 in HNE aSyn modification, we analyzed HNE-treated WT and H50Q/R aSyn by using MALDI-TOF MS. The analysis of GluC-digested aSyn variants showed that the H50Q/R mutations completely abolish the modification of residue 50. Importantly, substitution of H50 remarkably reduced the susceptibility of aSyn to HNE modification, indicating that H50 is the initial and most reactive residue of aSyn for HNE addition. We have previously shown that HNE-modified aSyn is more prone to form soluble oligomers [19]. Here, we observed significantly reduced formation of oligomers in H50 mutant aSyn compared to WT aSyn by using complementary assays for the analysis of oligomerization, i.e. SEC for the detection of soluble oligomers and SDS-PAGE for the detection of SDS-resistant oligomers. These findings suggest that H50 not only increases the reactivity of aSyn to HNE addition, but also plays a crucial role in HNE-induced aSyn oligomerization.

Based on previous findings showing that HNE modification increases the cytotoxicity of extracellularly applied aSyn [19-21] and that extracellular application of HNE causes HNE modification of intracellularly expressed aSyn [22], we hypothesized that overexpression of aSyn increases the susceptibility of cells to HNE due to the formation of intracellular H50-dependent HNE-aSyn modification. To verify this hypothesis, we overexpressed WT and H50Q/R aSyn in H4 cells and compared cell susceptibility to HNE by using two complementary methods, i.e. MTS viability assay and flow cytometric analysis. Our results indicate that the substitution of H50, the most reactive residue for HNE modification, reduces the susceptibility of aSyn overexpressing cells to HNE-induced toxicity. Thus, our experiments not only provide evidence that H50 plays a crucial role for HNE-mediated modification and oligomerization of aSyn, but also suggest its implication in HNE-induced cytotoxicity, probably due to HNE modification of intracellular aSyn at the H50 residue.

The identification of the novel PD-associated aSyn H50Q mutation provides evidence for the relevance of aSyn H50 not only in sporadic but also in monogenic PD. Thus, we asked whether the H50 mutations intrinsically potentiate aSyn pathology, despite reducing HNE-mediated aSyn aggregation and toxicity, thereby supporting the pathogenicity of the novel reported H50Q mutation. We analyzed the effects of H50 mutations on aSyn aggregation both *in vitro* using recombinant human aSyn, and in aSyn overexpressing cells. Analysis of the formation of amyloid fibrils in agitated aSyn samples using ThT assay demonstrated that H50Q mutation significantly increases the fibrillization of aSyn compared to WT aSyn, which is consistent with recent *in vitro* studies [27-29]. In contrast to a recent study [30], H50R did not significantly reduce fibrillization compared to WT aSyn in our experiments, likely due to different conditions for the fibrillization experiments (e.g. starting concentration of monomeric aSyn and buffer composition). In our study, the salt concentration in the aggregation buffer influenced the impact of the H50R aSyn mutation on fibrillization. Under physiological salt concentrations, H50R aSyn also showed higher fibrillization levels than WT aSyn, although less pronounced than H50Q aSyn. Consistently, we detected aSyn H50Q/R in the first high density fractions of SDGC, analogous to amyloid fibrils. These findings indicate that H50Q/R mutations intrinsically increase the propensity of aSyn to form larger aggregates. Yet, accumulating evidence suggests that small aggregation intermediates, especially oligomeric aSyn species, may be more toxic than large amyloid fibrils [31-33]. Therefore, in addition to fibrillization, we investigated the formation of oligomers. We did not detect soluble oligomers in unmodified recombinant WT aSyn and H50Q/R mutants by applying SEC. However, we were able to show that H50Q/R mutations, particularly the H50Q mutation, increased the formation of soluble aSyn oligomers in the presence of a nitrating agent, indicating that H50 mutant aSyn is more prone to form soluble oligomers under oxidative stress, characterized by accumulating ROS/RNS. Recently, it was shown that the H50Q mutation leads to a chemical shift within the C-terminal region (residue 135-140) of aSyn [30], affecting tyrosine 136, one target site for nitration [19]. Thus, H50 mutation may directly influence the accessibility or reactivity of this tyrosine residue to ROS/RNS, and consequently increase oligomerization propensity of aSyn in response to oxidative stress.

To validate our *in vitro* observation concerning the influence of H50 mutations on aSyn aggregation, we analyzed the impact of these mutations on intracellularly overexpressed aSyn in H4 cells. In a very recent study, in which the oligomerization of various aSyn mutants/variants was examined in HEK cells by using Bimolecular Fluorescence Complementation (BiFC) assay, no significant differences between WT and H50Q aSyn have been detected [34]. Interestingly, we observed an increase in H50Q aSyn immunosignal in higher density fractions of SDGC, suggesting an enhanced formation of oligomeric aSyn species in H4 cells. Taken together, our *in vitro* and cell-based experiments suggest that H50Q mutation intrinsically increases aggregation propensity of aSyn when compared to WT. Yet, while H50Q aSyn tends to form larger fibrillar aggregates *in vitro* as revealed by ThT and SDGC analysis, intracellularly overexpressed H50Q aSyn is more prone to the formation of oligomeric species as indicated by SDGC analysis. This discrepancy may be attributed to the presence of cellular factors that impact the aggregation kinetics of aSyn in cells, which is in accordance to our *in vitro* finding that additional treatment of aSyn with a nitrating agent increases the propensity of H50 mutant aSyn to form oligomers. Moreover, protein degradation pathways may also influence aSyn aggregation. In particular, we have recently shown that oligomeric and higher aggregated aSyn species are differently processed by autophagy as a major protein degradation pathway [33]. Therefore, intracelluar oligomerization of H50 mutant aSyn may be affected by autophagy associated pathways, which would be important to address in future studies.

To study the toxic effect of intracellularly expressed aSyn H50 mutants, we overexpressed WT and H50Q/R aSyn mutants in H4 cells by transient transfection. We generally observed a slight increase in cell damage due to H50Q/R mutations as measured either by ToxiLight toxicity or by MTS viability assay. In a more specific assessment of the direct effect of overexpressed aSyn on toxicity, we focused on aSyn overexpressing cells and analyzed the proportion of apoptotic cells among aSyn+ cells (aCasp3+/aSyn+). This specific cell death assay and the cell experimental conditions used in this study allowed us to detect a more pronounced toxic effect evoked by intracellularly overexpressed H50Q/R mutants, which was not revealed in other recent studies on pathological cellular effects of H50Q mutation [27,28]. We furthermore analyzed the influence of H50Q/R mutation on the H_2_O_2_-induced reduction of cellular viability. Our results showed that overexpression of H50Q/R mutant aSyn increases cellular susceptibility to H_2_O_2_ compared to WT aSyn. This finding is in agreement with a previous study observing this effect for H50Q aSyn in another cell model [27]. Using a H_2_O_2_ concentration range between 100 - 500 μM, we noticed that the increase in cellular susceptibility to oxidative stress due to H50Q/R mutation is especially marked at a lower H_2_O_2_ concentration (i.e. 100 μM) when compared to WT aSyn. The difference in cellular susceptibility between aSyn H50 mutants and WT aSyn declines with increasing H_2_O_2_ concentrations (i.e. 200 - 500 μM). This might be explained by the occurrence of global damage under strong oxidative stress conditions. Thus, our results suggest that the aSyn H50 mutation particularly increases cellular susceptibility at comparatively low levels of oxidative stress. Taking into account that oxidative stress conditions potentiate the formation of aSyn oligomers in H50 mutant aSyn (particularly H50Q aSyn) *in vitro*, our study indicates that the increased toxicity of H50Q/R mutant aSyn under oxidative stress might be associated with the formation of toxic oligomeric aSyn species.

Interestingly, H50Q/R mutant aSyn differentially influences cellular response to HNE and H_2_O_2_. Both stressors trigger cell death in a dose-dependent manner. However, while H50Q/R overexpressing cells appear to be more resistant to HNE than the WT aSyn overexpressing cells, they are more vulnerable to H_2_O_2_. These findings emphasize the role of H50 as an important HNE modification site in HNE-mediated toxicity, with particular relevance for sporadic PD. Additionally, the increased susceptibility to H_2_O_2_ due to H50 mutations suggests that the mechanistic role of the H50Q mutation in monogenic PD may rely on an altered neuronal response to general oxidative stress. To clarify whether H50 mutation generally increase cellular susceptibility to insults or whether this effect is specific to oxidative stress, further extensive studies are needed analyzing the impact of H50Q mutation under more specified oxidative stress and non-oxidative stress related conditions.

In this study, we substituted H50 by two amino acid residues with different chemical characteristics, i.e. the uncharged and polar glutamine and the basic arginine (like histidine). Both mutations impact aggregation behavior and toxicity of aSyn, although to a different extent. Thus, our results suggest that the effects of the PD-related H50Q mutation are attributed to the loss of a functional H50 residue rather than to a specific gain of function induced by the glutamine. Nevertheless, the H50R mutation appeared to be more toxic than the H50Q mutation, although H50R aSyn was less prone to aggregation than H50Q aSyn *in vitro,* and increased intracellular aggregation of H50R aSyn was not detectable by using SDGC. These interesting findings suggest that besides aggregation, other mechanisms affecting the physiological or pathological function of aSyn have to be considered when investigating H50-mutation dependent toxicity.

## Conclusion

**Figure 9:**
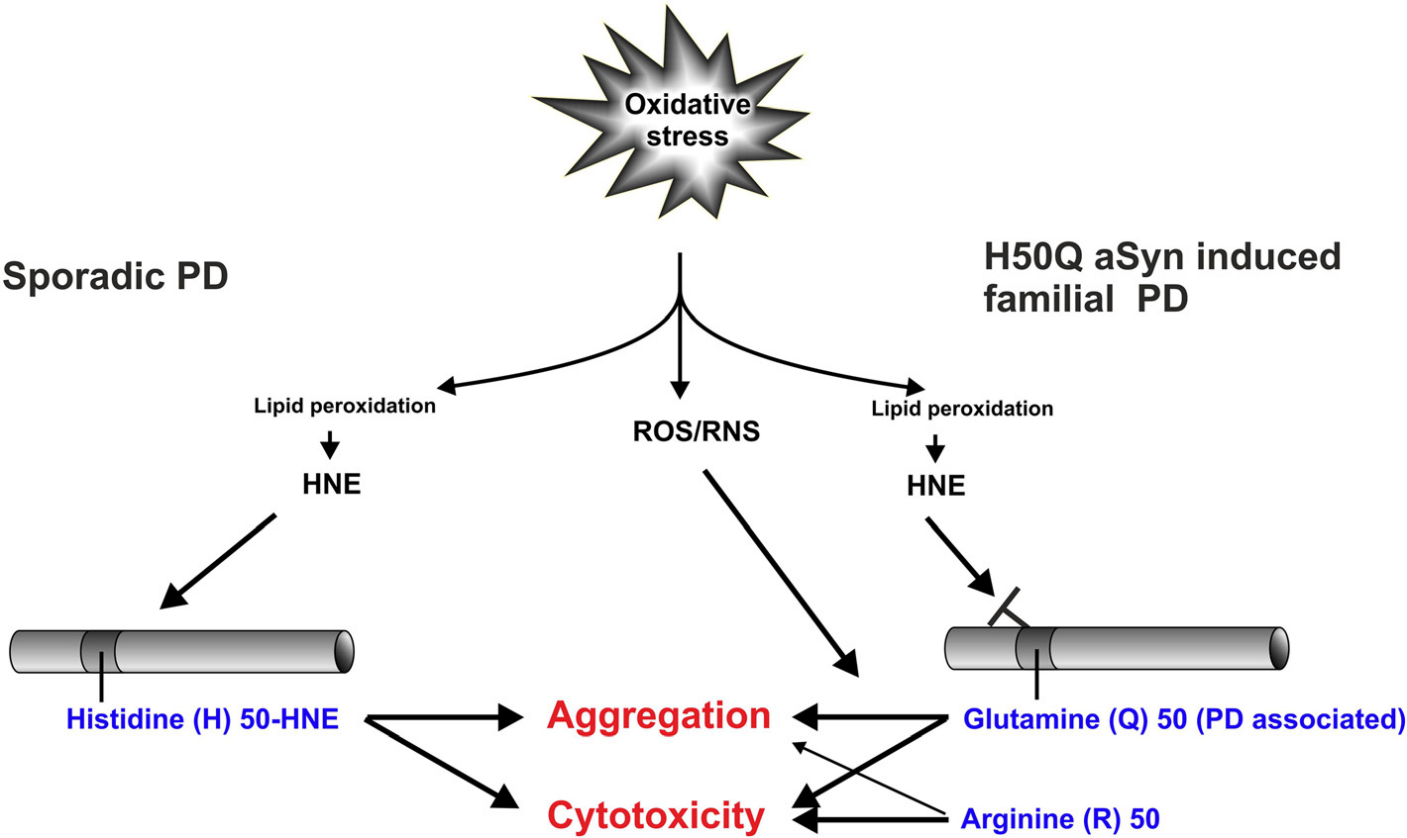
**Summary of the impact of aSyn H50 modification and mutation on aSyn pathology.**

Our data demonstrate that aSyn H50 alterations, either by oxidative stress-related HNE modification or by gene mutation, increase aggregation propensity and cytotoxicity of aSyn (Figure 9). Moreover, we reveal that oxidative stress not only triggers aSyn pathology by directly modifying H50 of WT aSyn via HNE addition, but also potentiates the pathological effects of the H50Q mutation. Although H50Q mutation diminishes the HNE-mediated effects on aSyn, it intrinsically increases aSyn aggregation and toxicity, and exacerbates the cellular susceptibility to oxidative stress, especially to ROS/RNS other than the lipid peroxidation product HNE. Thus, we provide novel insights into the mechanisms underlying oxidative stress-associated pathogenesis of sporadic and H50Q-associated monogenic PD.

## Methods

### Site-directed mutagenesis

The QuikChange® Site-directed Mutagenesis Kit (Agilent Technologies, Waldbronn, Germany) was applied to generate both aSyn H50Q/R mutations in the coding sequence of WT aSyn in human aSyn pcDNA3.1 and pT7-7 constructs according to the manufacturer’s protocol. The mutation-covering, complementary primer sequences 5′ G GAG GGA GTG GTG *CAG* GGT GTG GCA ACA G 3′ and 5′ C TGT TGC CAC ACC *CTG* CAC CAC TCC CTC C 3′, as well as 5′ G GAG GGA GTG GTG *CGT* GGT GTG GCA ACA G 3′ and 5′ C TGT TGC CAC ACC *ACG* CAC CAC TCC CTC C 3′ were used to generate aSyn H50Q and H50R mutations, respectively.

### Preparation of recombinant human aSyn and induction of PTMs

Constructs containing WT aSyn and aSyn H50 mutants were transformed in *E. coli* BL21 (DE3) pLys competent cells (Novagen, San Diego, CA, USA). The preparation and purification of recombinant WT and H50Q/R mutant aSyn were performed as previously described [19]. To induce nitration, recombinant aSyn of 70 μM was incubated with 5 mM tetranitromethane (TNM) in 30 mM Tris/HCl pH 7.4 at room temperature (RT) for 7 h. HNE-modified aSyn was generated by incubating aSyn with HNE concentrations ranging from 50 μM to 3000 μM in 30 mM Tris/HCl pH 7.4 at 37°C for 24 h.

### Mass spectrometry

Detection of HNE-modified aSyn was performed by matrix-assisted laser-desorption-ionization time-of-flight mass spectrometry (MALDI-TOF MS) as previously described [19]. Briefly, for the digestion with GluC endoproteinase (Roche Diagnostics GmbH, Penzberg, Germany) 3-5 μg recombinant aSyn and 500 ng GluC was dissolved in 50 mM NH_4_HCO_3_ and incubated overnight at RT. Full-length aSyn or GluC-digested protein samples were mixed with 0.1% trifluoroacetic acid (TFA) (v/v) and MALDI matrix 2,5-dihydroxyacetophenone, spotted on a stainless steel target, and measured by a Bruker Autoflex (Bruker Daltonik, Bremen, Germany). Positive ions were analyzed in reflector mode after acceleration by 20 kV. External calibration was performed using a peptide calibration standard (Bruker Daltonik). Each displayed mass spectrum was produced by five individual spectra, which were generated by 50 shots/individual spectrum recorded from several positions on a spot. Spectra were analyzed using Flex Analysis software (Bruker Daltonik). Mass of aSyn ions or GluC-digested aSyn fragments are given in the mass of singly charged [M + H]^+^ ions.

### Size exclusion chromatography

Size exclusion chromatography (SEC) was applied to assess soluble oligomeric aSyn. Prior to SEC, samples were centrifuged at 100000 g for 60 min. aSyn species were separated on a SuperdexTM 75 10/300 column (GE Healthcare, Freiburg, Germany) using 30 mM Tris/HCl, 0.2 M NaCl, pH 7.4 as an eluent at a flow rate of 0.5 ml/min and monitoring the UV absorbance at 280 nm. To ensure reproducibility between SEC runs, Gel Filtration Standard (Bio-Rad Laboratories, Munich, Germany) was used prior to each set of analysis.

### *In vitro* fibrillization of aSyn and Thioflavin T assay

WT and H50Q/R mutant aSyn of 70 μM in 30 mM Tris/HCl pH 7.4 (100 μl per sample) were incubated in a 96-well plate and agitated at 200 rpm on a Controlled Environmental Incubator Shaker (New Brunswick Scientific INC, New Brunswick, NJ, USA) at 37°C for 7 d in the presence of glass beads with a diameter of 2 mm. For the detection of aSyn fibrils, 6 μg of agitated aSyn was diluted in 30 mM Tris/HCl pH 7.4 containing 20 μM ThT (Sigma-Aldrich, Taufkirchen, Germany) and incubated for 10 min at 300 rpm and RT in the dark. The fluorescence intensities were measured in a Varian Cary Eclipse fluorescence spectrophotometer (Agilent Technologies, Waldbronn, Germany) with an excitation at 445 nm and an emission at 480 nm. For quantification, values were normalized to the fluorescence intensity of non-agitated WT aSyn.

### Electron microscopy

Electron microscopy (EM) was performed to analyze fibrillization of WT and H50 mutant aSyn. For negative staining, 10 μl of each agitated aSyn sample at a concentration of 70 μM were adsorbed onto glow-discharged carbon-coated Formvar grids (Electron Microscopy Sciences, Hatfield, PA, USA), incubated for 20 min, washed with distilled water, and then stained with a filtered 3% aqueous uranyl acetate solution for 5 min. After another washing step, grids were dried and analyzed with a transmission electron microscope (LEO 906E; Carl Zeiss, Oberkochen, Germany).

### Cell culture, calcium-phosphate transfection, and cell treatment

H4 neuroglioma cells of human origin (ATCC, HTB-148) were maintained in Opti-MEM + GlutaMAX (51985-042, Invitrogen, Darmstadt, Germany) supplemented with 10% fetal calf serum (FCS; 10270-106, Invitrogen, Darmstadt, Germany) at 37°C. 24 h prior transfection cells were plated in 24-well plates (3.5 × 10^4^ cells/well) or in 6-well plates (2 × 10^5^ cells/well) and cultured in 0.5 ml or 2 ml medium, respectively. Calcium-phosphate transfection was performed as previously described [35]. Equimolar ratios of pcDNA3.1 plasmids encoding human WT, H50Q, and H50R aSyn were transfected under a cytomegalovirus promoter. Mock transfection without adding plasmid DNA served as control. The transfection efficiency was controlled by using flow cytometry. For the treatment experiments, H4 cells were exposed to different concentrations of HNE (50 – 3000 μM, Cayman Chemical Company, Ann Arbor, MI, USA) or H_2_O_2_ (100 - 500 μM, Merck, Darmstadt, Germany) 24 h after transfection for 12 h (HNE) or 24 h (H_2_O_2_), respectively. The stock solution of 64 mM HNE was prepared in ethanol. The stock solutions of HNE and H_2_O_2_ were freshly diluted in culture medium prior to treatment. Vehicle controls (ethanol or culture medium) were prepared in accordance to the highest HNE and H_2_O_2_ concentration used for the treatments.

### Detection of aSyn transfection efficiency and expression levels via flow cytometry

H4 cells were seeded in 24-well plates (3.5 × 10^4^ cells per well), transfected with pcDNA3.1 expression vectors encoding WT and H50Q/R mutant aSyn, and harvested 24 h after transfection. Cells were washed with PBS and fixed by using a 1:4 dilution of Fixation/Permeabilization Concentrate (eBioscience, Frankfurt, Germany) in Fixation/Permeabilization Diluent (eBioscience). Cells were washed with Permeabilization Buffer (eBioscience) and blocked for 15 min by using 10 μl of FcR Blocking Reagent (human, Miltenyi Biotech, Bergisch Gladbach, Germany). Detection of aSyn was performed by applying a rat anti-human aSyn primary antibody (1 h, 1:400, Enzo Life Sciences, ALX-804-258-L001, Lörrach, Germany) and an Alexa488-labeled donkey anti-rat secondary antibody (1 h, 1:800, A21208, Invitrogen, Darmstadt, Germany) diluted in Permeabilization Buffer. 20000 cells were detected to analyze aSyn transfection efficiency and the mean intensity of the aSyn signal via flow cytometry with a CyFlowR Space (Partec, Münster, Germany) and the FloMax 2.81 analysis and quantification software. The forward and sideward scatter signal was used to determine the population of single cells used for measurements. The cut-off fluorescence intensity for defining aSyn expressing cells was set by measuring mock transfected cells treated with the same staining protocol.

### Assessment of toxicity and cell viability

Toxicity of aSyn transfected H4 cells was analyzed in 24-well plates by ToxiLight enzyme activity assay for membrane integrity (Lonza, Basel, Switzerland), and MTS viability assays (Promega, Mannheim, Germany) according to the manufacturer’s protocol, 36 h and 48 h after transfection, respectively.

In order to measure apoptotic cell death, activated (cleaved) Caspase 3 positive cells were assessed by ICC. For this, H4 cells were plated on 13 mm glass coverslips prior to transfection. 36 h after transfection, cells were fixed with 4% paraformaldehyde for 15 min. Cells were washed with Tris buffered saline (TBS, pH 7.4) and blocked with fish skin gelatin buffer (FSGB) containing 50 mM Tris/HCl, pH 7.4, 1% BSA, 0.2% fish skin gelatin, and 0.1% Triton-X 100 for 1 h at RT. Cells were incubated with primary rat anti-human aSyn (1:250, Enzo Life Sciences, ALX-804-258-L001, Lörrach, Germany) and rabbit anti-aCasp3 (1:500, 9661, Cell Signaling Technology, Danvers, MA, USA) antibodies overnight at 4°C. After washing, Alexa 488-labeled donkey anti-rat secondary antibody (1:1000, A21208, Invitrogen, Darmstadt, Germany) and Alexa 568-labeled donkey anti-rabbit secondary antibody (1:1000, A10042, Invitrogen) were applied for 1 h at RT. Nuclei were counterstained with 4′6′-diamidino-2-phenylindol (DAPI, 1:10000, D8417, Sigma-Aldrich, Steinheim, Germany) for 15 min. After washing, coverslips were mounted by using Prolong Antifade reagent (P36930, Invitrogen). The ratio of cells positive for aCasp3 (aCasp3+) among aSyn positive (aSyn+) cells was assessed in accordance to a systematic, random counting procedure [19]. For image acquisition, Axio Imager M2 microscope combined with an AxioCam MRm camera (Carl Zeiss AG, Jena, Germany) with the same settings and exposure times within each independent experiment was used. Six images of each coverslip were randomly selected at 20 × magnification to enable the analysis of at least 200 aSyn positive cells per independent experiment. aSyn+ and aCasp3+ cells were scored based on the presence of immunostaining compared to the background staining of the corresponding controls.

### SDS-PAGE and Western blot

For Western blot (WB) of recombinant aSyn, 0.5 μg aSyn was mixed with one volume of SDS sample buffer (0.125 M Tris/HCl pH 6.8, 4% SDS, 20% glycerol), separated on 15% SDS-PAGE, and blotted onto nitrocellulose membranes (Millipore, Darmstadt, Germany). The blots were probed with a mouse anti-aSyn primary antibody (Syn-1, 1:2000, BD Transduction Laboratories, San Diego, CA, USA) or a rabbit anti-aSyn antibody (SNCA antibody, 1:2000, Proteintech Europe, Manchester, UK). While Syn-1 was generated using aSyn fragment amino acids 15-123 as antigen, SNCA antibody was raised against full-length aSyn. The nitrocellulose membranes were subsequently probed with secondary goat-anti-mouse antibody or goat anti-rabbit antibody coupled to horseradish peroxidase (1:10000 Dianova, Hamburg, Germany). For the detection of proteins, membranes were incubated with the SuperSignal West Pico or Femto Sensitivity Substrate™ (Thermo Scientific Rockford, lL, USA). Immunoblots were visualized by VersaDoc gel imaging system (BioRad, Munich, Germany).

### Sucrose density gradient centrifugation and dot blot

Sucrose density gradient centrifugation (SDGC) was performed as previously described [36] with minor modifications. Briefly, a continuous 10 - 30% sucrose gradient (30 ml) in 25 mM Tris-HCl, pH 7.4, 0.2 M NaCl was prepared on top of the 60% sucrose cushion (4 ml). For analyzing recombinant aSyn samples, 30 μg of aSyn was loaded on top of the sucrose gradient. For analyzing aggregation of cellular expressed aSyn, H4 cells were seeded in a 6 well plate (2 × 10^5^ cells per well) for transfection. Cells from 3 wells were collected 36 h after transfection via scraping in ice-cold PBS containing protease/phosphatase inhibitors and pooled for SDGC analysis. Subsequently, cell pellets were homogenized in 50 mM Tris/HCl pH 7.4 buffer containing 150 mM NaCl, 2 mM EDTA, 1% (v/v) NP-40, 0.1% (w/v) SDS, and complete mini protease inhibitors cocktail (Roche Diagnostics GmbH) in a Potter dounce homogenizer at 4°C. The lysates were loaded on top of the sucrose gradient. After centrifugation in a Beckman L-70 Ultracentrifuge with a SW-28 rotor (Beckman Coulter, Brea, CA, USA) at 26000 rpm for 18 h at 4°C, 22 sucrose fractions with 1.5 ml each were collected. 400 μl of each fraction were mixed with 100 μl methanol and spotted on a nitrocellulose membrane. After blocking the nitrocellulose membranes, aSyn was visualized using a mouse anti-aSyn primary antibody (Syn-1, 1:2000, BD Transduction Laboratories), and a HRP-conjugated goat anti-mouse secondary antibody (1:10000, Dianova). For the detection of aSyn in SDGC fractions, membranes were incubated with the SuperSignal West Pico or Femto Sensitivity Substrate™ (Thermo Scientific).

### Statistical analysis

Statistical analyses were performed using GraphPad Prism (GraphPad Software, San Diego, CA, USA). All numeric results are reported as mean + standard error of the mean (SEM) and represent data from a minimum of three independent experiments unless otherwise stated. Significant differences are depicted in the figures by graphical representation. p < 0.05 was considered as significant = *. p < 0.01 = **. p < 0.001 = ***.

## Additional file

Additional file 1: Figure S1. Determination of transfection efficiency by flow cytometry. **Figure S2.** Analysis of the susceptibility of WT and H50 mutant aSyn overexpressing cells to HNE by flow cytometry.

## Abbreviations

aCasp3: Activated (cleaved) Caspase 3
aSyn: Alpha synuclein
BCA: Bicinchonic acid
BSA: Bovine serum albumin
DAPI: 4′,6′-Diamidino-2-phenylindol
EDTA: Ethylenediaminetetraacetic acid
EM: Electron microscopy
FCS: Fetal calf serum
FSGB: Fish skin gelatin buffer
HNE: 4-hydroxy-2-nonenal
H_2_O_2_: Hydrogen peroxide
HRP: Horseradish peroxidase
ICC: Immunocytochemistry
LB: Lewy bodies
MALDI TOF: Matrix-assisted laser desorption/ionization time-of-flight
MS: Mass spectrometry
MTS: 3-(4,5-dimethyl thiazol-2-yl)-5-(3-carboxymethoxyphenyl)-2-(4-sulfophenyl)-2H-tetrazolium
m/z: Mass to charge ratio
PD: Parkinson’s disease
PTM: Posttranslational modification
RNS: Reactive nitrogen species
ROS: Reactive oxygen species
RT: Room temperature
SDGC: Sucrose density gradient centrifugation
SEC: Size exclusion chromatography
SNCA: Alpha synuclein gene
TFA: Trifluoroacetic acid
ThT: Thioflavin T
TNM: Tetranitromethane
WB: Western blot
WT: Wild type
