# Supplementary material for: Posttranslational modification and mutation of histidine 50 trigger alpha synuclein aggregation and toxicity
Source: Mol Neurodegener. 2015 Mar 11;10:8. doi: 10.1186/s13024-015-0004-0 (PMC4365527; doi:10.1186/s13024-015-0004-0)

**Additional file 1**

**Figure S1. Determination of transfection efficiency by flow cytometry.**

The proportion of cells transfected with WT and H50 mutant aSyn was determined via flow cytometry 24h after transfection by aSyn staining as shown in Figure 3B and C. In addition, we applied GFP and aSyn (GFP/aSyn) cotransfection of H4 cells for a fast quantification of transfected cells via flow cytometry. To this end, plasmids encoding aSyn and GFP were mixed prior to generation of the calcium phosphate precipitates, leading to cotransfection of aSyn and GFP in H4 cells. We observed that transfection of H4 cells with a mixture of 9:1 pcDNA3.1 plasmids encoding aSyn and GFP resulted in simultaneous overexpression of both aSyn and GFP in almost all transfected cells as detected by ICC (Figure S1A). Detection of aSyn transfected cells either evaluated by GFP fluorescence signal in GFP/aSyn cotransfected cells or detected by aSyn immunosignal in aSyn transfected cells shows that both methods are equally precise in determination of proportion of aSyn transfected cells (Figure 3B, and Figure S1B & S1C). For the GFP/aSyn cotransfection, cells were seeded in 6 well plates or 24 well plates. 100000 or 20000 cells from 6 well plates or 24 well plates, respectively, were measured 17 h, 24 h or 36 h after transfection. Transfection efficiency was evaluated by counting GFP+ cells with flow cytometry as exemplarily shown in Figure S1B. H4 cells transfected with WT, H50Q, and H50R aSyn vectors showed a comparable transfection rate 17 h, 24 h, and 36 h after transfection (Figure S1C, two-way ANOVA Bonferroni multiple comparisons).

**Figure S1**


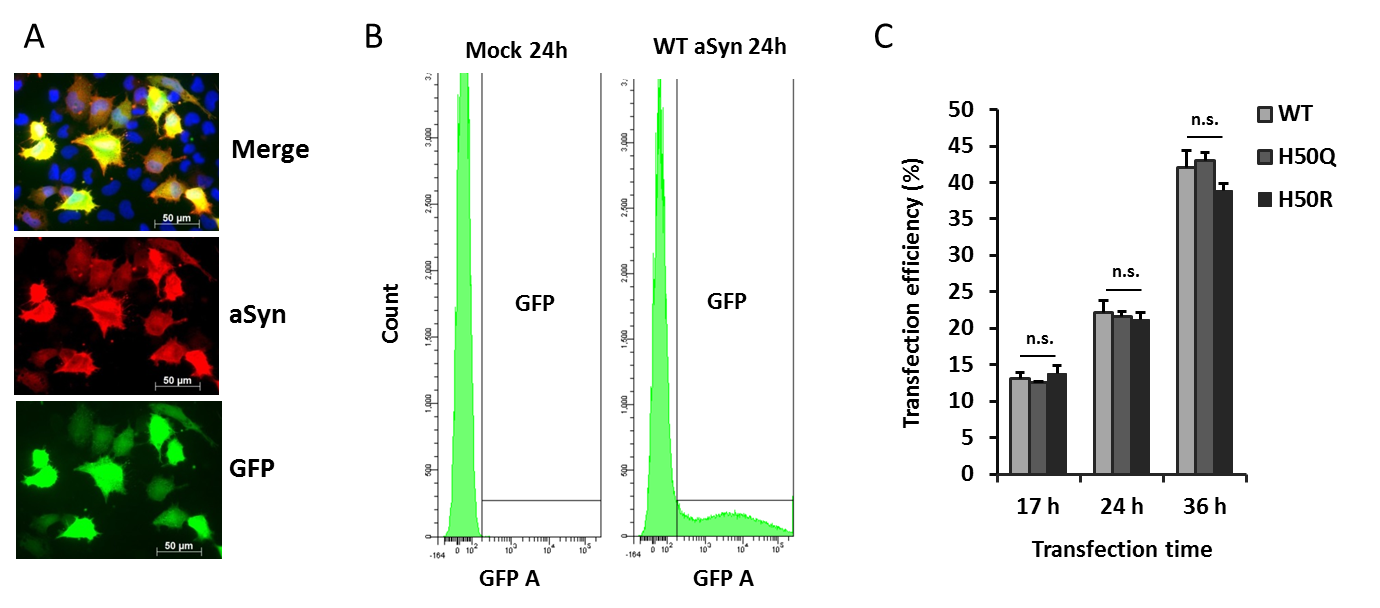


**Figure S2. Analysis of the susceptibility of WT and H50 mutant aSyn overexpressing cells to HNE by flow cytometry.**

The proportion of WT and H50Q/R aSyn transfected H4 cells was assessed 12 h after HNE administration by GFP/aSyn cotransfection and flow cytometry as described above (Figure S1). As shown in Figure S2, HNE led to a concentration-dependent proportional reduction of transfected cells in comparison to the corresponding vehicle-treated cells. This finding suggests that aSyn overexpressing cells are in general more vulnerable to HNE than non-overexpressing cells. Yet, while for H50 mutant aSyn transfected cells a significant reduction was only detectable after applying 60 µM HNE, WT aSyn expressing cells already showed a significant reduction at 40 µM HNE when compared to vehicle treated cells as determined by one-sample t-tests. Moreover, we analyzed the results with two-way ANOVA Bonferroni multiple comparisons and detected a significant difference between WT and H50R aSyn transfected cells at 40 µM HNE. These results are in line with the results of the MTS assays (Figure 3) and underline the importance of H50 for HNE-induced toxicity.

**Figure S2**


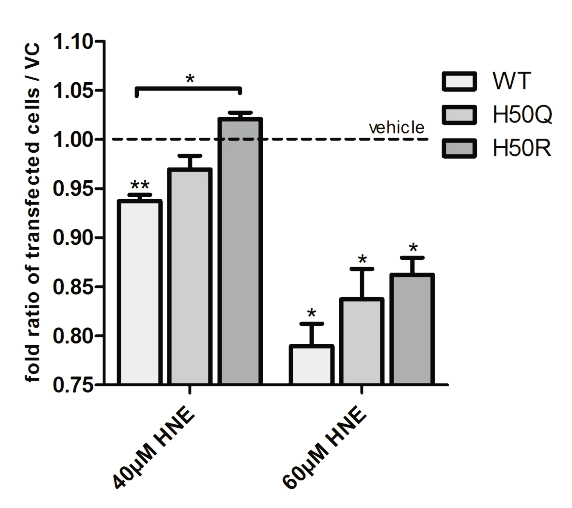

Supplement: Additional file 1: Figure S1. — Determination of transfection efficiency by flow cytometry. Figure S2. Analysis of the susceptibility of WT and H50 mutant aSyn overexpressing cells to HNE by flow cytometry. [file 13024_2015_4_MOESM1_ESM.docx]
